# Diffusion Tensor Magnetic Resonance Imaging for Differentiating Multiple System Atrophy Cerebellar Type and Spinocerebellar Ataxia Type 3

**DOI:** 10.3390/brainsci9120354

**Published:** 2019-12-03

**Authors:** Chi-Wen Jao, Bing-Wen Soong, Chao-Wen Huang, Chien-An Duan, Chih-Chun Wu, Yu-Te Wu, Po-Shan Wang

**Affiliations:** 1 Institute of Biophotonics, National Yang-Ming University, Taipei 11221, Taiwan; c3665810@ms24.hinet (C.-W.J.); poloplay_1988@hotmail.com (C.-W.H.); andyduan5109@hotmail.com (C.-A.D.); 2 Brain Research Center, National Yang-Ming University, Taipei 11221, Taiwan; 3 Department of Neurology, Shin-Kong Wu Ho Su Memorial Hospital, Taipei 11101, Taiwan; 4 Taipei Neuroscience Institute, Taipei Medical University, Taipei 11041, Taiwan; bwsoong@gmail.com; 5 Department of Neurology, Taipei Veterans General Hospital, Taipei 11221, Taiwan; 6 School of Medicine, National Yang-Ming University, Taipei 11221, Taiwan; 7 Department of Radiology, Taipei Veterans General Hospital, Taipei 11221, Taiwan; 8 The Neurological Institute, Taipei Municipal Gan-Dau Hospital, Taipei 11261, Taiwan

**Keywords:** DTI, SCA3, MAS-C, cerebellum, K-means clustering

## Abstract

Multiple system atrophy cerebellar type (MSA-C) and spinocerebellar ataxia type 3 (SCA3) demonstrate similar manifestations, including ataxia, pyramidal and extrapyramidal signs, as well as atrophy and signal intensity changes in the cerebellum and brainstem. MSA-C and SCA3 cannot be clinically differentiated through T1-weighted magnetic resonance imaging (MRI) alone; therefore, clinical consensus criteria and genetic testing are also required. Here, we used diffusion tensor imaging (DTI) to measure water molecular diffusion of white matter and investigate the difference between MSA-C and SCA3. Four measurements were calculated from DTI images, including fractional anisotropy (FA), axial diffusivity (AD), radial diffusivity (RD), and mean diffusivity (MD). Fifteen patients with MSA-C, 15 patients with SCA3, and 30 healthy individuals participated in this study. Both patient groups demonstrated a significantly decreased FA but a significantly increased AD, RD, and MD in the cerebello-ponto-cerebral tracts. Moreover, patients with SCA3 demonstrated a significant decrease in FA but more significant increases in AD, RD, and MD in the cerebello-cerebral tracts than patients with MSAC. Our results may suggest that FA and MD can be effectively used for differentiating SCA3 and MSA-C, both of which are cerebellar ataxias and have many common atrophied regions in the cerebral and cerebellar cortex.

## 1. Introduction

Cerebellar ataxias can be categorized into two subgroups: hereditary or sporadic. Spinocerebellar ataxia type 3 (SCA3), also known as Machado–Joseph disease, is the most common hereditary cerebellar ataxia type [1,2]. SCA3 is caused by the expansion of a CAG repeat in the ataxin-3 gene [1,2]. The etiology of the cerebellar atrophy is currently known to involve chromosomal abnormalities and familial genetic predisposition [2]. Its symptoms include slow progressive ataxia, lisp, dysphagia, unclear writing font, delicate hand movement, unstable gait, and other movement control-related symptoms [3]. The cerebellum is one of the most affected areas in patients with SCA3; additionally, atrophy or neuronal loss in the cerebellar cortex, vermis, peduncles, and deep nuclei have been reported in some neuropathological studies [2,4,5]. MSA-C is characterized by two or more neurological degeneration symptoms, such as pyramidal, extrapyramidal, cerebellar, and autonomic system symptoms [6,7,8]. The fundamental lesions of MSA-C present in the arcuate, pontine, inferior olivary, and pontobulbar nuclei and the cerebellar cortex [6,7,8]. Many investigators have reported that the main pathological changes of MSA-C are the loss of neurons in the ventral portion of the pons, inferior olives, and cerebellar cortex. The fundamental lesions of MSA-C occur in the arcuate, pontine, inferior olivary, and pontobulbar nuclei and the cerebellar cortex [9,10,11].

Diffusion tensor imaging (DTI) can facilitate the visualization and characterization of white matter (WM) fascicli in two and three dimensions [12]. Since the introduction of this methodology in 1994, it has been used to study the WM architecture and integrity in normal brains, diseased brains (e.g., brains of patients with multiple sclerosis, stroke, dementia, and schizophrenia), and brains of older people [12]. In DTI analysis, tract-based spatial statistics (TBSS) is the most applied method [13]. TBSS can provide information on anatomical connectivity in the brain by measuring the anisotropic diffusion of water in WM tracts [13]. MSA-C and SCA3 are both neurodegenerative diseases with dominant WM atrophy [8,14,15]. Previous studies showed patients with SCA3 demonstrate decreases in fractional anisotropy (FA) in the areas of cerebellum and brainstem, but increases in radial diffusivity (RD) in the cerebellum, brainstem, thalamus, and frontal and temporal lobes [16]. However, the results of TBSS studies have not been completely consistent [15,16]. Another TBSS study identified widespread FA reduction in the bilateral cerebral-frontal, -parietal, -temporal, and -occipital WM; cerebellar WM; the thalamus; and the brainstem in patients with SCA3. Moreover, mean diffusivity (MD) increases were detected in a similar, widely overlapping pattern in bilateral cerebral-frontal, -parietal, -temporal, and -occipital WM; cerebellar WM; the thalamus; and the brainstem [15]. Regarding MSA-C, a TBSS study detected significant decreases in FA in the bilateral corticospinal tract (CST) and right anterior thalamic radiation (ATR) but increases in RD in the bilateral CST [17]. Another study reported an increase in the apparent diffusion coefficient in the middle-cerebellar peduncles and cerebellum but decreased FA in the pyramidal tract, middle-cerebellar peduncles, and WM of the cerebellum in patients with MSA-C compared with healthy individuals [18].

Several studies have indicated that patients with SCA3 or MSA-C exhibit the same atrophied regions, such as those in the cerebrum, pons, vermis, frontal gyrus, temporal gyrus, and basal ganglia [13,14,15,16,17,18,19,20,21,22,23]. Moreover, they share similar manifestations, including ataxia, pyramidal and extrapyramidal signs [24]. Clinically, MSA-C and SCA3 cannot be differentiated based on the clinical symptoms or T1-weighted magnetic resonance imaging (MRI) images [25]. Hence, an accurate diagnosis of SCA3 and MSA-C requires genetic testing [24]. Changes in the DTI images of patients with SCA3 or MSA-C have been reported previously [15,16,26]. However, most of the reports have mainly focused on the cerebellum and brainstem of patients with SCA3 or MSA-C and only described the differences between patients with MSA-C or SCA3 and the healthy controls. Thus far, the investigation of DTI on the whole brain (cerebrum, cerebellum, and brainstem) for differences between SCA3 and MSA-C is still unexplored. Thus, we used DTI to quantify the WM of patients with MSA-C and patients with SCA3 and aim to evaluate the efficiency of DTI in detecting differences between SCA3 and MSA-C.

## 2. Materials and Methods

### 2.1. Participants

The study procedures were performed in accordance with the Declaration of Helsinki, with the approval of by the Institutional Review Board of Taipei Veterans General Hospital (Supplementary material 1: Ethical Approval). Table 1 lists the demographic data of all participants. Fifteen patients with MSA-C, 15 patients with SCA3 patients, and 30 healthy subjects (control group) participated in this study. Firstly, the severity of ataxia was graded as follows: (I) Walking without assistance, (II) walking with partial assistance, (III) needing assistance walking, (IV) needing assistance standing, and (V) bedridden [27]. Diagnoses of MSA were made according to the established guidelines [22]. All patients with MSA met the second consensus criteria for a clinical diagnosis of probable MSA [22]. According to the clinical symptoms, patients were categorized into MSA-C. Moreover, genetic testing was performed for each patient. The CAG repeat length of each SCA3 patient was determined by polymerase chain reaction, as described in our previous study [28], and confirmed they were patients with SCA3.All participants provided written informed consent, approved by the Ethics Committee of Taipei Veterans General Hospital.

### 2.2. DTI Data Collection

Axial brain T1-weighted, 3-dimensional, fast-spoiled, gradient-recalled acquisition of steady state images and DTI images of the entire cerebrum and cerebellum of all the participants were acquired using a 1.5T Vision Siemens MRI scanner (Erlangen, Germany) at Taipei Veterans General Hospital. The image parameters of the T1-weighted images were TR = 7.5 ms, TE = 2.1 ms, flip angle = 15°, slice thickness = 1.5 mm, FOV = 256 × 256 mm^2^, and number of slices = 124 (with a 1.5-mm gap). DTI images of all participants were obtained using a fast spin-echo EPI sequence with 14 diffusion-weighted volumes. These diffusion-weighted volumes included 13 volumes with gradients applied along 13 independent orientations (b = 1000 s/mm^2^), and 1 volume was obtained without diffusion weighting. Each volume comprised 65 continuous axial slices with a slice thickness of 3 mm that covered the entire cerebrum and cerebellum. The image parameters were TR = 17,000 ms, TE = 70.2 ms, flip angle = 90°, FOV = 128 × 128 mm^2^, and the voxel size of each DTI image slice was 2.5 × 2.5 × 3 mm .

By applying specific magnetic field gradients, MRI can be sensitized to the random, thermally driven motion (i.e., Brownian motion) of water molecules in the direction of the field gradient. The diffusion is anisotropic in the fiber tracts, the axonal membranes and myelin sheaths, the direction of which is mainly parallel to the fiber tracts, present barriers to the motion of the water molecules. In particular, the tensor model of diffusion consists of a 3 × 3 matrix derived from the diffusivity measurements in at least six noncollinear directions. Mathematically, there are three eigenvectors and three eigenvalues in each diffusion tensor matrix of each voxel. The eigenvectors ($\epsilon_{1}$, $\epsilon_{2},\;\epsilon_{3}$) represent the major, medium, and minor principal axes of the ellipsoid, respectively, and the eigenvalues (λ_1_, λ_2_, λ_3_) represent the diffusivities in these three directions, respectively.

Four important measurements are commonly used in the DTI analysis: FA, MD, AD, and RD. Table 2 summarizes the formula of each of the measurements. FA, the most widely used anisotropy measure, is often considered a measure of the WM integrity [29]. FA can measure the anisotropic fraction of the diffusion; in other words, it measures the deviation of the tensor ellipsoid’s shape from that of a perfect sphere [30]. FA is a scalar value lying between zero and one and describes the degree of anisotropy of a diffusion process where it is considered to reflect fiber density. If FA = 0, diffusion is isotropic (spherical), whereas if FA = 1, diffusion occurs along one axis only and is completely restricted along all the other axes.

**Figure 1:**
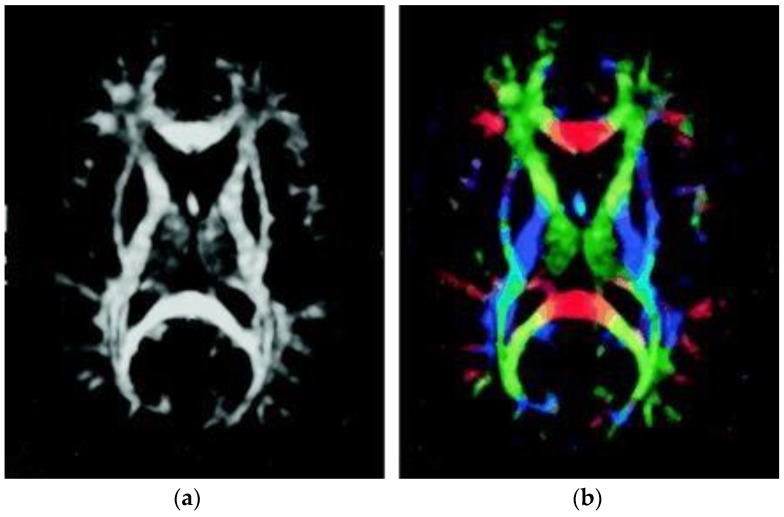
FA map from a control group participant. (**a**) FA map without directional information. (**b**) Combined FA and directional map. Colors represent the direction of the fiber tract: red, left–right; green, anterior–posterior; blue, superior–inferior.

Figure 1 illustrates the FA map of one control individual. Gray matter, consisting of the neuronal cell bodies, appears dark; by contrast, WM, containing relatively very few cell bodies and being chiefly composed of long-range myelinated axon tracts, demonstrates a higher signal and has higher FA due to restriction by the myelin sheaths. Figure 1a illustrates the FA map without directional information. In Figure 1b, the colors represent the direction of the fiber tract; red represents the link between left and right, green the link between the anterior and posterior regions, and blue the link between the superior and inferior regions of the brain.

MD, an inverse measure of the membrane density, is sensitive to cellularity, edema, and necrosis. AD tends to be variable in WM changes and pathology and has been reported to increase with the maturation of the WM tracts [31]. RD increases with dysmyelination of WM; changes in the axonal diameters or density may also influence RD [32,33].

### 2.3. Diffusion Tensor Images Preprocessing

**Figure 2:**
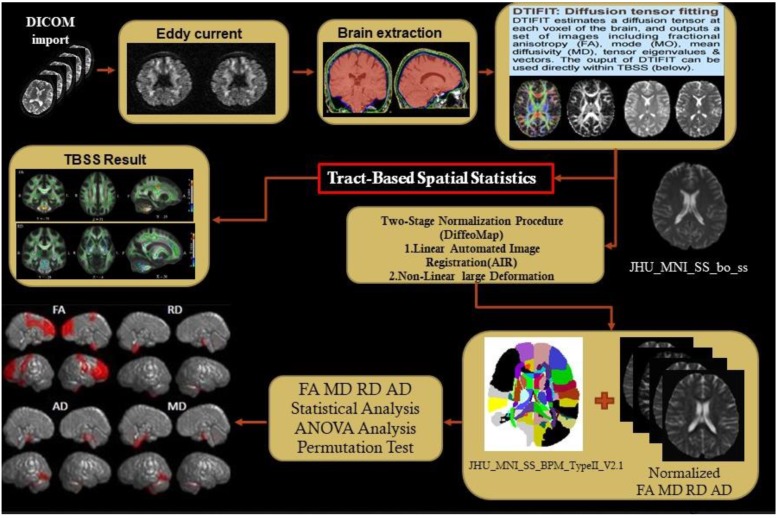
Flowchart of DTI analysis.

Figure 2 illustrates the flow of the DTI analysis. We used two software packages to preprocess the diffusion tensor image: (1) FSL (FMRIB Software Library, FMRIB, Oxford, UK; https://fsl.fmrib.ox.ac.uk/fsl/fslwiki) to perform the eddy current correction, Skull stripping (Brain Extraction Tool), and compute four DTI measurements and (2) Diffeomap implanted in MRI studio was used to normalize the DTI images. After the normalization of the DTI images, TBSS was used to improve the sensitivity, objectivity, and interpretability of analysis of the multi-subject diffusion images [34]. There were four core steps in TBSS: (1) Select a common registration target image, namely, FMRIB58_FA in this study, and then use the nonlinear registration method to register the preprocessed images with the target image, (2) average all post registration FA images and then compress the averaged FA map by using the non-maximum suppression method to produce the averaged FA skeleton map [35], (3) identify the maximum FA in the perpendicular direction on the averaged FA skeleton and then use all the maximum FA values to constitute an FA skeleton of the participants, and (4) use a threshold to filter out the lower averaged FA and the larger variation. The threshold FA was set as 0.2 for separating gray matter and WM to facilitate the final voxel level statistical analysis.

### 2.4. TBSS and Structural Parcellation

**Figure 3:**
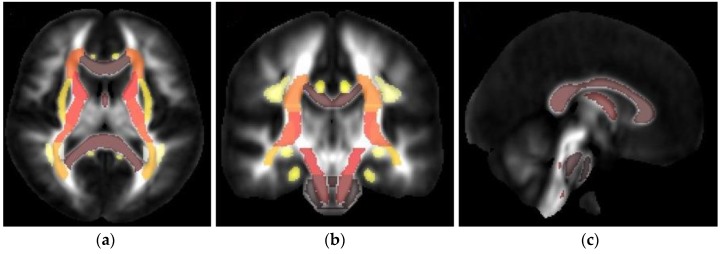
Tract-based spatial statistics (TBSS) result of DTI in the regions of interest in the JHU-ICBM-labels atlas: (**a**) axial view, (**b**) coronal, and (**c**) sagittal view. Colors represent the direction of the fiber tract: dark-brown, left–right; yellow and light brown, anterior–posterior; and red, superior–inferior.

The TBSS-processed DTI images were then parcellated using the JHU DTI-based white-matter atlases, consisting of only the WM labels (Supplementary Table S1). Based on the atlas, labeled WM areas were extracted for the TBSS statistical image. Figure 3 illustrates the TBSS result of DTI in the regions of interest in the JHU-ICBM-labels atlas of one control participant: axial, coronal, and sagittal views.

### 2.5. Statistical Analysis

One-way ANOVA was used to evaluate significant differences between the control, SCA3, and MSA-C groups for the FA, AD, RD, and MD of each investigated WM region. The significance of the results was corrected according to the false discovery rate (threshold = 0.05). The general linear model (GLM) [36] was used while adjusting for age and sex as nuisance covariates to regress the sex and age effects on the FA, AD, RD, and MD in each group. After ANOVA analysis, the post-hoc test was performed to see the effect of the means different conditions [37].

To statistically compare the differences in FA, AD, RD, and MD between all groups, a permutation test was conducted [38]. In the process, a significant *p* value was computed for the control, SCA3, and MAS-C groups. For instance, to test the null hypothesis that the DTI differences between the control and SCA3 group occurred by chance, we randomly reassigned the patients with SCA3 and healthy controls into two groups and recomputed the statistical difference in their DTI values for each randomized group. These randomized simulations and recalculation processes were repeated 2000 times. The 95th percentile points of each distribution of the 2000 simulations were used as critical values in a two-sample one-tailed *t* test to reject the null hypothesis with a type I error probability of 0.05.

### 2.6. K-Means Clustering

Clustering is an unsupervised method for partitioning or grouping a given set of patterns into disjoint clusters. Similarity plays a dominant factor for clustering. If the patterns are alike then they will be grouped into the same cluster, and patterns belonging to two different clusters are different. Clustering has been a widely studied problem in a variety of application domains including neural networks, AI, and statistics. K-means clustering, proposed by MacQueen in 1967, is a method commonly used to automatically partition observations into k clusters in which each observation belongs to the cluster with the nearest mean, serving as a prototype of the cluster [39]. The k-means method has been shown to be effective in producing good clustering results for many practical applications [40]. The following steps summarize the operations of k-Means [40]. Initialize k cluster centers. In practice, this can be done by either randomly selecting k center. 1) Random generation of k center points. 2) Calculate the distance between each observation and the cluster centers. 3) Assign each data to the cluster whose distance from its center is minimum of all the cluster centers. 4) Re-compute the positions of the k centers as the cluster mean. 5) Re-compute the distance between each data point and the newly computed centers. 6) Repeat steps 3 and 4 until the assignment of data points does not change (data points do not move). In this study, we used DTI parameters as features in k-means clustering to group participants, and aim to evaluate the efficiency of DTI in detecting differences between SCA3 and MSA-C.

## 3. Results

### 3.1. Patients with SCA3 or MSA-C Demonstrated Significant Decrease in FA and Increase in AD, RD, and MD

**Figure 4:**
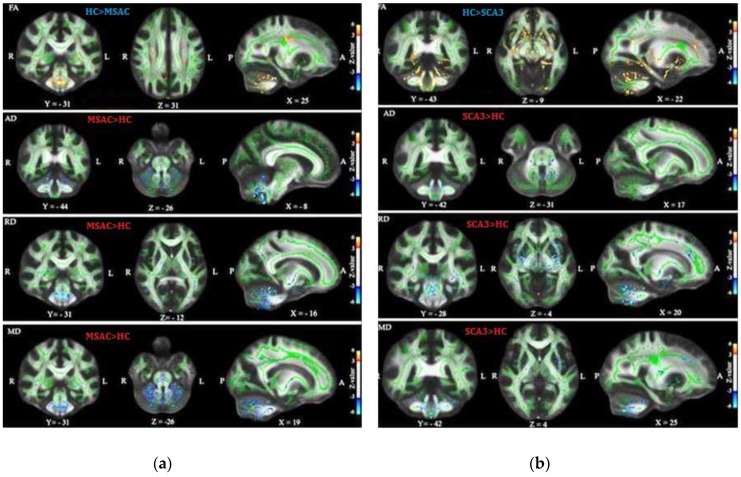
One-way ANOVA statistical analysis of FA, RD, AD, and MD of DTI between groups (**a**) Results between health control group (HC) and MSAC (**b**) Results between HC and SCA3.

Table 3(a) summarizes the tracts that revealed significant changes in FA, RD, AD, and MD between the control and SCA3 groups, and Table 3(b) the significant change tracts between the control and MSA-C groups. Figure 4 illustrates the one-way ANOVA statistical analysis of FA, RD, AD and MD of DTI between health control and MSA-C (Figure 4a), and Figure 4b illustrates the results between health control and SCA3 groups. FA decreased in the MSA-C and SCA3 groups and AD, RD, and MD were increased mainly in the cerebello-ponto-cerebral loops. Compared with the control group, the SCA3 group had significantly decreased FA and increased RD and MD in the inferior-cerebellar peduncle, middle-cerebellar peduncle, corticospinal tract, posterior limb of internal capsule, anterior corona radiates, and external capsule. The MSA-C group demonstrated significantly decreased FA and increased RD and MD in the superior-cerebellar peduncle, corticospinal tract, middle-cerebellar peduncle, and posterior limb of internal capsule compared with the control group. Compared with patients with MSA-C, patients with SCA3 demonstrated significantly decreased FA, mainly in the frontal lobe, but larger values of AD, RD, and MD in the genu of the corpus callosum and the superior cerebellar peduncle in the cerebello-cerebral tracts.

### 3.2. Patients with SCA3 Demonstrated Significant Decrease in FA and Increases in RD, AD, and MD

**Figure 5:**
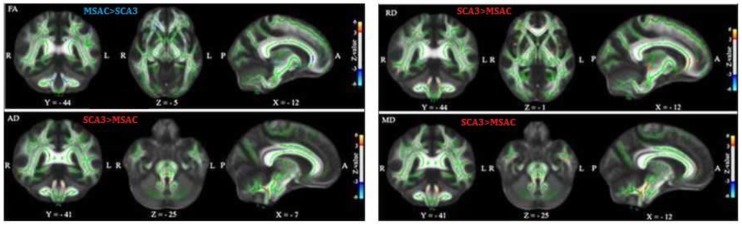
TBSS result in patients with MSA-C and patients with SCA3. The general linear model (GLM) model for age regression was used, two-sample *t* test and permutation test to investigate the significant differences in FA, AD, RD, and MD between the control and SCA3 groups. The red and blue color voxels are the regions with significantly decreased *z* value (>3) and those with significantly increased *z* value (<−3) in patients with SCA3, respectively.

Figure 5 depicts the TBSS analysis results between MSAC and SCA3 groups. We used the GLM model for age regression and a two-sample *t* test and permutation test to investigate the significant differences in FA, AD, RD, and MD between groups. In each sub plot, the red color voxels illustrate the regions of significant decrease with *z* value > 3 and the blue color voxels illustrate the regions of significant increase with *z* value < −3. Compared with the control group, patients with SCA3 or MSA-C demonstrated significant decrease in FA and increases in AD, RD, and MD in the cerebello-ponto-cerebral tracts. Moreover, patients with SCA3 patients demonstrated the most significant decrease in FA and the most significant increases in AD, RD, and MD in the cerebello-cerebral tracts. The detailed regions with significant differences for TBSS results of FA, AD, RD, and MD of each group are listed in Supplementary Tables S2–S4.

**Figure 6:**
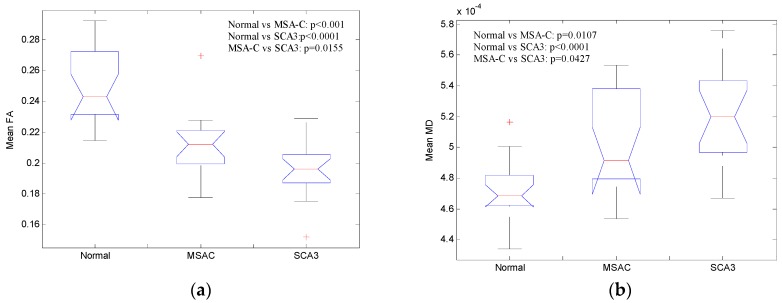
Mean (**a**) FA of and (**b**) MD of cerebral and cerebellar white matter (WM) for the three groups.

Figure 6a illustrates the mean FA of cerebral and cerebellar WM and Figure 6b illustrates the mean MD of cerebral and cerebellar WM in all three groups. Patients from both MSA-C and SCA3 showed significantly decreased FA (Normal vs MSA-C: *p* < 0.001, Normal vs SCA3: *p* < 0.0001, MSAC vs SCA3: *p* = 0.0155, verified by post-hoc test) and increased MD (Normal vs MSA-C: *p* = 0.0107, Normal vs SCA3: *p* < 0.0001, MSAC vs SCA3: *p* = 0.0427, verified by post-hoc test) of cerebral and cerebellar WM, and the patients with SCA3 revealed the most significantly decreased FA and increased MD among all the groups. These results suggest that both MSA-C and SCA3 are WM-dominant diseases, and patients with SCA3 may suffer more WM lesion than patients with MSA-C.

## 4. Discussion

In this study, we used noninvasive DTI to explore WM integrity in the control, SCA3, and MSA-C groups and computed the FA, AD, RD, and MD in each group. The ANOVA results revealed significantly differences in the FA, AD, RD, and MD of various regions including the frontal and temporal lobes and cerebellum. In the TBSS analysis, we found increased FA and decreased AD, RD, and MD in many of the WM regions, and these results were consistent with the previous SCA3 [15] and MSA-C [18,41] TBSS studies.

TBSS studies on patients with SCA3 have demonstrated that FA reduction is widespread in the bilateral cerebral-frontal, -parietal, -temporal, and -occipital WM; cerebellar WM; the thalamus, and the brainstem [42]. Moreover, MD increases were detected in a similar, widely overlapping pattern as in FA, particularly in bilateral cerebral-frontal, -parietal, -temporal, and -occipital WM; cerebellar WM; the thalamus, and the brainstem [42]. These studies showed the increase of RD as the main driving force underlying the increase in MD. Furthermore, we found similar regions (Figure 5 and Supplementary Tables S3–S5) mainly in the brainstem, cerebellum and frontal and temporal lobes, with decreased FA and increased AD, RD, and MD in patients with SCA3. Studies have reported that the increase in MD and reduction in FA are mostly collocated, suggesting a common process underlying these changes in the diffusion profile [43]. Moreover, Beaulieu demonstrated that the reduction of FA, in contrast to an increase in MD alone, has been associated with axonal loss as a potential mechanism [44]. The cognitive impairments in patients with SCA3, including verbal and visual memory deficits, visuospatial dysfunction, and executive dysfunction, have been described previously [22,45]. These neuropsychological abnormalities in SCA3 may be associated with cerebellar cognitive affective syndrome (CCAS) [46,47,48]. CCAS potentially involves the dysfunction of circuits connecting the cerebellum to the prefrontal lobe. Superior parietal, superior temporal and limbic cortices have been reported [47]. This hypothesis was supported by the two published VBM and free-surfer studies [49,50]. In the current study, we found that FA decreased and RD increased in the corticospinal tract, cortico-bulbar (superior corona radiate, posterior limb of internal capsule, and cerebral peduncle) tract, and cortico-ponto-cerebellar tract (middle cerebellum peduncle) in patients with SCA3. According to WM TBSS results, we may hypothesize that the movement disorder in SCA3 is caused by not only cerebellar injury but also cerebral atrophy [49]. A possible explanation is that precentral neuronal loss might disrupt the corticospinal, cortico-bulbar, and cortico-ponto-cerebellar tracts and result in the loss of fibers. This hypothesis is supported by our results and by recent TBSS studies in patients with SCA3 [50].

In a previous VBM study, patients with MSA-C demonstrated atrophy in the cerebellum, insular cortex, fusiform gyrus, inferior orbitofrontal gyrus, superior temporal gyrus, and caudate nucleus [19]. In our current TBSS results, we found that the patients with MSA-C demonstrated decreased FA in the external capsule, superior-cerebellar peduncle, medial lemniscus, retrolenticular part of internal capsule, fornix (cres)/stria terminalis, middle-cerebellar peduncle, posterior limb of internal capsule, corticospinal tract, inferior-cerebellar peduncle, cerebral peduncle, and superior corona radiate. In addition, these patients exhibited increased RD in the inferior-cerebellar peduncle, superior-cerebellar peduncle, cerebral peduncle, corticospinal tract, external capsule, and middle-cerebellar peduncle. Compared with the results of a previous TBSS study on patients with MSA-C, which reported significantly decreased FA in bilateral the CST and right ATR, and increased RD in the bilateral CST [51], our results revealed more areas with significantly decreased FA and increased RD. In addition, our results presented increased AD in the superior and inferior cerebellar peduncles and increased MD in inferior-cerebellar peduncle, superior-cerebellar peduncle, corticospinal tract, middle-cerebellar peduncle, and posterior limb of the internal capsule. In humans, the cerebellum plays an important role in motor control; it is also involved in some cognitive functions, such as attention and language, as well as in working memory and visuospatial analysis [52]. The cortico-ponto-cerebellar tract, composed of the corticopontine tract, links the cerebellum to the prefrontal, sensorimotor cortices, prefrontal cortex, and temporal lobe [53]. Thus, damage to the cerebellum or cerebello-ponto-cerebral tract can result in CCAS and ataxia. The pathological changes, such as neuronal loss, astrogliosis, and loss of myelinated fiber, and deposition of glial cytoplasmic inclusions were distributed in the cortical regions, including frontal insular cortex, parietal lobe, and temporal lobe that have been demonstrated in the previous postmortem studies [17,54,55] In the current study, we found decreased FA but increased RD and MD in cortical spinal cord tracts and middle cerebellum peduncle. Thus, the disease potentially affects cognitive deficits in MSA-C. Moreover, in the current study, compared with patients with MSA-C, patients with SCA3 presented decreased FA in the genu of corpus callosum, superior-cerebellar peduncle, inferior-cerebellar peduncle, and anterior corona radiate; moreover, in addition to increased AD in the superior-cerebellar peduncle, we noted increased RD in the genu of corpus callosum, superior-cerebellar peduncle, inferior-cerebellar peduncle, anterior corona radiate, and external capsule and increased MD in the superior-cerebellar peduncle and external capsule. Thus, patients with SCA3 may have more severe clinical symptoms than do those with MSA-C.

**Figure 7:**
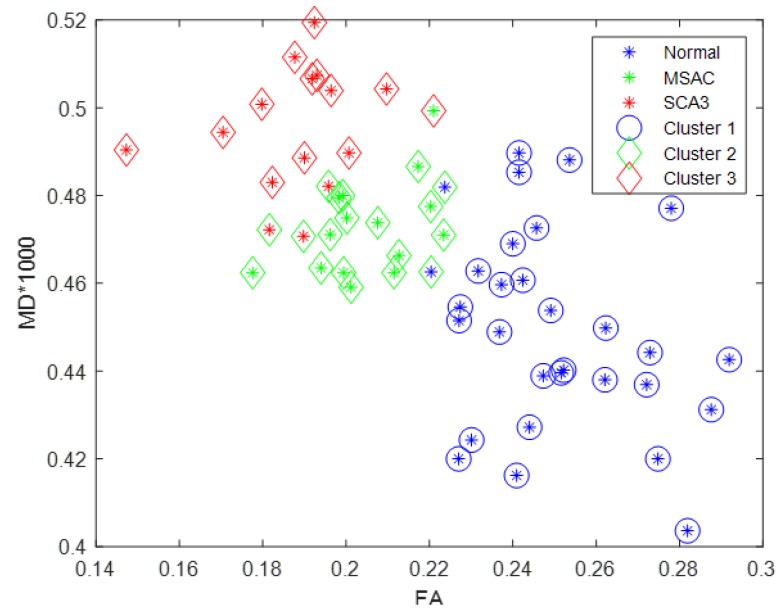
Scatter plot of rescaled MD versus FA for all participants. Blue, green, red stars denote the control participants, patients with MSA-C, and patients with SCA3, respectively; the three groups can be linearly separated by K-means clustering with few overlaps.

We subsequently rescaled mean MD of cerebral and cerebellar WM of each participant by 1000, such that this value (between zero and one) was comparable with mean FA of cerebral and cerebellar WM. Figure 7 illustrates the scatter plot of FA (*x* axis) versus MD (*y* axis) for all participants by K-means clustering method. The control, MSA-C, and SCA3 groups were disproportionately distributed and could be separated linearly. Therefore, the concurrent use of FA and MD can be an effective feature for differentiating SCA3 and MSA-C cases, even though SCA3 and MSA-C are both cerebellar ataxias and share many common atrophied regions in the cerebral and cerebellar cortices.

However, this study has some limitations. First, because SCA3 and MSA-C are rare diseases, collecting data from a sufficiently large group of patients for our study would have been time-consuming and difficult. Thus, because of the relatively small sample size of our study, the statistical power of our results might be low. Second, we did not correlate the TBSS results with the clinical measurements, such as the mini–mental state examination (MMSE) or scale of the assessment and rating of ataxia.

## 5. Conclusions

We investigated the changes in DTI images in patients with SCA3 and patients with MSA-C. Both patients with SCA3 and patients with MSA-C demonstrated significant decreases in FA and significant increases in AD, RD, and MD in the cerebello-ponto-cerebral tracts. Patients with SCA3 demonstrated lower in FA and higher in AD, RD, and MD in the cerebello-cerebral tracts compared with the patients with MSA-C. The results suggested that patients with SCA3 may suffer more severe clinical symptoms than patients with MSA-C revealed.

## Figures and Tables

**Table 1:** Participant demographics. SCA3: Spinocerebellar ataxia type 3; MAS-C: Multiple system atrophy cerebellar type.

Group	Subjects	Sex (M/F)	Age
Control	30	15/15	46.57 ± 16.2
SCA3	15	7/8	43.8 ± 14.8
MAS-C	15	7/8	56.2 ± 6.62

**Table 2:** Four important diffusion tensor imaging (DTI) measurements.

Measurement	Formula
Fractional Anisotropy (FA)	$\sqrt{\frac{3}{2}}\sqrt{\frac{{\left(\lambda_{1}-\bar{\lambda }\right)}^{2}+{\left(\lambda_{2}-\bar{\lambda }\right)}^{2}+{\left(\lambda_{3}-\bar{\lambda }\right)}^{2}}{\lambda_{1}^{2}+\lambda_{2}^{2}+\lambda_{3}^{2}}}$ where $\bar{\lambda }$ = ($\lambda_{1}$ + $\lambda_{2}$ + $\lambda_{3}$)/3
Mean Diffusivity (MD)	(λ_1_ + λ_2_ + λ_3_ )/3
Axial Diffusivity (AD)	λ_1_
Radial Diffusivity (RD)	(λ_2_ + λ_3_ )/2

**Table 3:** ANOVA analysis.

**a. ANOVA analysis pass FDR between HC and SCA3 (α value = 0.05)**					
**Tract name**	**HC v.s. SCA3**		**FA *p*-value**	**RD *p*-value**	**MD *p*-value**
ATRL			3.00E−08	0.000578	0.003286
ATRR			1.27E−08	1.01E−05	9.19E−05
CCF			1.88E−08	0.000136	0.001167
CgLL			3.12E−06	1.40E−07	6.29E−07
CgLR			1.72E−06	4.22E−09	1.48E−08
CgUL			1.37E−08	1.58E−06	1.72E−05
CgUR			3.75E−08	1.47E−05	0.00016
CSTL	FA 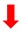 RD,MD 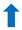		1.10E-05	0.000329	0.001618
CSTR			5.45E-05	5.33E−05	0.000192
IFOL			3.34E−07	0.000712	0.005848
IFOR			4.00E−07	0.000138	0.001238
ILFL			1.57E−05	0.001387	0.006487
ILFR			3.42E−06	0.000251	0.00152
SLFBL			3.66E−06	3.21E−05	0.000186
SLFBR			2.98E−06	4.22E−05	0.000215
UNCL			1.03E−09	0.000147	0.00245
UNCR			5.67E−09	4.27E−05	0.000682
**b. ANOVA analysis pass FDR between NC and MSAC (α value = 0.05)**					
**Tract name**	**HC v.s. MSAC**		**FA *p*-value**	**RD *p*-value**	**MD *p*-value**
ATRL			5.24E−05	0.000236	0.000688
ATRR			0.000164	1.94E−05	7.84E−05
CCF			0.01287	0.014828	0.032523
CgLL			7.03E−09	2.12E−09	1.88E−08
CgLR			1.32E−09	2.00E−11	2.67E−10
CgUL			7.05E−06	4.26E−07	2.77E−06
CgUR			3.49E−05	9.77E−05	0.00042
CSTL	FA 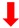 RD,MD 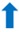		0.010202	0.006932	0.011976
CSTR			0.008366	0.004881	0.009157
IFOL			0.002727	0.003176	0.008229
IFOR			0.003212	0.005515	0.013653
ILFL			0.000557	0.000261	0.000811
ILFR			8.61E−05	0.000509	0.001974
SLFBL			0.000247	0.000689	0.001565
SLFBR			0.00264	0.006788	0.012182
UNCL			0.003825	0.001234	0.003551
UNCR			0.008912	0.00186	0.00538

## Supplementary Materials

The following are available online at https://www.mdpi.com/2076-3425/9/12/354/s1, Supplementary Material 1: Ethical Approval, Table S1: JhuMniSSBPM TypeIIV2.1LabelLookupTable, Table S2: The TBSS statistical results of significantly different regions between Control and SCA3 in FA, AD, RD and MD, Table S3: The TBSS statistical results of significantly different regions between Control and MSA-C in FA, AD, RD and MD, Table S4: The TBSS statistical results of significantly different regions between SCA3 and MSA-C in FA, AD, RD and MD.
